# A Study on Nurse Manager Competency Model of Tertiary General Hospitals in China

**DOI:** 10.3390/ijerph19148513

**Published:** 2022-07-12

**Authors:** Sitong Wang, Jiayu Tong, Yang Wang, Dan Zhang

**Affiliations:** 1 Institute for Hospital Management, Tsinghua Shenzhen International Graduate School, Tsinghua University, Shenzhen 518055, China; wang-st20@mails.tsinghua.edu.cn (S.W.); tongjy20@mails.tsinghua.edu.cn (J.T.); 2 Hainan Xinyang Education Training Co., Ltd., Haikou 570100, China; cassie_sssit@163.com

**Keywords:** competency model, tertiary general hospital, nurse manager, selection

## Abstract

(1) Background: The nurse manager competency model is widely used in hospitals in the management of nursing human resources. This study aimed to construct a scientific and practical model of nurse manager competency, which can be used as a tool by hospitals to select nurse managers. (2) Methods: The nurses’ competency indicators were first collected through a literature review and behavioral event interview, and the preliminary screening of competency elements was based on the Delphi method. Then, questionnaires and statistical analyses were used to determine the elements of the model. The reliability was determined using Cronbach’s alpha. Factor analysis was used to delineate the dimensions of the model. (3) Results: The results of the factor analysis show that the 22 competency elements were grouped into the four dimensions of leadership and management ability, personal traits, professional quality, and professional ability. The Cronbach’s alpha coefficients of the four dimensions ranged from 0.745 to 0.885, which indicated a high level of reliability. The result of the factor analysis indicated a high structural validity and fitting effect. (4) Conclusions: Our study indicates that the nurse manager competency model of tertiary general hospitals is an instrument with fair reliability and validity that fully reflects the characteristics of nurses in Chinese public hospitals.

## 1. Introduction

As the principle of healthcare delivery, medical staff are directly related to the ability and quality of health services, as well as health outcomes [1,2]. Nurses are indispensable in all healthcare settings because they care for patients who suffer from illnesses and ensure their quality of life. Nurse managers, as their name implies, are unit administrative leaders responsible for the quality of patient care and using both professional knowledge and administrative power in clinical practice [3,4]. The key roles of nurse managers focus on technical, professional, administrative, and fiscal categories. Proper staffing/scheduling, hiring/recruiting, developing staff competencies, role-modeling, retaining staff, and coaching/mentoring professional roles are included in the professional category. The administrative roles are often described as being the types of relational roles that market/champion the hospital, pay attention to the Joint Commission on Accreditation of Healthcare Organizations standards/paperwork reports, cover for other departments, manage admissions, and find beds [5]. Anthony found that the roles of nurse managers include clinical nursing activities, implementing evidence-based practices and organizational change, budget planning, and ensuring employee satisfaction and retention [6,7]. As a result of healthcare system reform, technological innovation, a growing elderly population, and a growing medical burden, a more complex healthcare work environment has placed greater demands on the competence of nurse managers [8,9]. Several studies show that nurse managers need to resolve interpersonal conflicts among staff and promote nurse relationship building [5]. It has been demonstrated that nurse managers play a key role in the establishment of a healthy workplace, the recruitment and retention of clinical nurses, and improvements in the quality of care and patient safety [10,11]. As the main body of nursing management, nurse managers are required to master higher levels of education and experience compared with other nurses [12]. Tertiary general hospitals are associated with the highest healthcare-delivery competence [13], so nurse managers of tertiary general hospitals are expected to be more proficient in both skills and tasks compared with those of second-tier hospitals. Existing studies suggest that nurse managers of tertiary general hospitals need to be responsible for work organization and administrative management, participate in service-quality supervision and managing the funding budget of the hospital, and improve the efficiency of nursing human resource management and quality of nursing work [14], which makes a nurse manager’s role unique compared with roles in other healthcare settings.

Most nurse managers in Chinese tertiary general hospitals are selected from clinical nurses based on their explicit characteristics such as their education background, professional knowledge, and nursing skills, without paying much attention to implicit factors such as responsibility, cooperation, and leadership [15,16]. However, these implicit characteristics, being undervalued, are inclined to exert considerable influence on nurse managers’ working performance, so the current selection methods are not scientific and rigorous enough. Under this circumstance, a comprehensive nurse manager competency model is needed to measure a nurse manager’s knowledge and skills, as well as their value orientation, competitive consciousness, desire for knowledge, and other potential characteristics. Such a model can act as a selection standard for nurse managers, and hospitals can select candidates with more potential and who fit the job requirements more closely [17].

The methods for constructing the nurse manager competency model are mainly divided into qualitative and quantitative research, ranging from the Delphi method, job analysis, and the behavioral-event-interview method to the questionnaire-survey method [18]. Liu constructed a model through the Delphi method including three dimensions: service and help, management characteristics, and personal efficacy and cognition [19]. Supamanee and Sherman constructed models through the interview method, including six dimensions: self-transcendence, interpersonal communication ability, financial management ability, human resource management, empathy, and systematic thinking ability [20,21,22]. McCarthy and Pillay constructed models by using questionnaire surveys, including professional skills, coordination and communication ability, human resource management, adaptability, innovation ability, and decision-making ability [23,24]. McCarthy, Pillay, and Dai constructed models through nuclear inspection tables and the behavioral-event-interview method, including professional learning ability, thinking ability, leadership, communication skills, talent training, team spirit, initiative, self-confidence, and dedication spirit [23,24,25]. Tian constructed a model through focus-group discussions and the online-survey method. The model consisted of five key competency features of high-level community health service management, including interpersonal coordination, communication ability, business resources, administrative and management capability, knowledge of the healthcare environment and organization ability, change leadership and management skills, and the ability to make decisions based on facts [26].

Despite the above model constructed by domestic and foreign scholars, there are few studies on the competency of nurse managers in tertiary general hospitals [19]. The existing model studies have not distinguished the research objects, and whether the results are applicable to tertiary general hospitals has not been verified. For example, Dazhi W. applied the established nurse managers’ competency model to the quality assessment of nurse managers through in-tray tests and leaderless group discussions, and drew a conclusion that the assessment results were quite consistent with the reality [27]. Xianghua X. established a model through behavioral event interviews and questionnaire surveys, designed a training course for nurses according to the competency elements in the model, and evaluated the training effect for head nurses by using the competency assessment questionnaire, which showed that the scores of head nurses in the competency assessment questionnaire improved significantly after training [28]. Some of the existing models only include competency indicators, and the model structure is incomplete, resulting in a lack of connection between the theoretical framework and practical applications, and its practicality needs to be further explored [29]. Due to different national conditions and cultures, the application of foreign competency-model methods may not be applicable to China. Therefore, competency research suitable for China’s local conditions is expected to be developed.

## 2. Materials and Methods

### 2.1. Sample

In this study, survey samples were selected through two-stage sampling. In the first stage, the study chose Beijing, Jilin, and Hainan based on economic competitiveness through judgement sampling. In the second stage, through simple random sampling, 3 tertiary hospitals in Beijing, 2 tertiary hospitals in Changchun, and 2 tertiary hospitals in Haikou were selected, and 600 questionnaires were distributed.

### 2.2. Study Design

The research started to obtain nurse managers’ competencies in two ways, which are a literature review and behavioral event interview, and then integrated all the obtained competencies to be preliminary ones for further screening to establish the model. The Delphi method was conducted twice, and the ratio of full scores, mean, and coefficient-of-variation method was adopted to define the number, names, and definitions of competencies; then, the questionnaires containing these competencies were distributed to nurse managers from tertiary general hospitals for further competency screening by virtue of the ratio of full scores, mean, and coefficient-of-variation method. Factor analysis was employed to delineate the dimensions of the competencies, and the competency system was subsequentially tested by using Cronbach’s alpha and the structural equation model. An analytic hierarchy process was consequently utilized for the weights of each dimension and competency. The nurse manager competency model was eventually constructed through the above procedures.

### 2.3. Study Procedures

The selection of the nurse manager competency model elements for the questionnaire was based on a literature review, a behavioral event interview, and the Delphi method. First, through literature analysis, 10 representative nurse manager competency models were selected as the source of preliminary elements and 20 competency elements were extracted.

The behavioral event interview is an open and behavioral retrospective exploration technique combining the critical event method and thematic apperception test. Through interviews, descriptions of successful and unsuccessful events during the interviewees’ tenure were collected. Based on these descriptions, we could find detailed behaviors that affected the performance in target positions, and then collect, analyze, and code the collected specific events and behaviors to obtain competency elements [30]. In the behavioral event interview, 14 nurse managers from three tertiary general hospitals in Beijing were selected. All of the 14 nurse managers had more than 2 years of experience in nursing management and had excellent performance appraisals. Moreover, to ensure representativeness, nurse managers were selected from internal medicine, surgery, obstetrics and gynecology, pediatrics, and traditional Chinese medicine departments. Meetings were held to determine the selection criteria for the interview subjects and the compilation and modification of “the Competency Coding Dictionary for Nurse Managers in Tertiary General Hospitals”. By converting the record into text and analyzing key words, we extracted 23 competency elements. After combining the elements from the literature analysis and behavioral event interview, removing the same elements, a total of 31 nurse manager competency elements were obtained.

Second, we screened model elements using the two-round Delphi method with a panel of 20 experts. These experts were selected nationwide according to their education backgrounds, professional titles, years of experience, majors, and positions. All the experts had more than 5 years of working experience in nursing management. The average age was (44.53 ± 10.19) years old, the average working years in nursing management was (18.84 ± 9.11) years, about 60% had obtained a master’s degree or above, and about 70% had obtained titles of deputy senior or above.

The recovery rates for the first and second rounds of consultation were 95% and 100%, respectively. The results showed that the authority degree coefficient for all the factors in the two rounds was higher than 0.8, suggesting that the results of the experts’ evaluation had high reliability. The Kendall coordination coefficient for all the factors was statistically significant (*p* < 0.05), suggesting that the results of two rounds had high validity. According to the 31 elements collected, the questionnaire on the importance of competency elements was compiled, and two rounds of expert consultation were conducted. After two rounds of Delphi, 27 factors were retained.

Finally, this study finally determined the competency elements through a questionnaire survey of nurse managers in tertiary general hospitals. After two rounds of Delphi, we compiled the Questionnaire on the Importance of Competency Elements for Nurse Managers of Tertiary General Hospitals. The questionnaire asked the participants to rate the importance of each factor on a 5-point Likert scale, ranging from “very important” (5 points) to “very unimportant” (1 point).

Before the formal questionnaire survey began, we conducted a small-scale pre-survey to assess the reliability and validity of the questionnaire. The pre-survey investigated 145 nurse managers from 3 tertiary general hospitals in Beijing. A total of 139 questionnaires were collected. After receiving the completed questionnaires, a preprocessing step was applied to remove incomplete or invalid data. The exclusion criteria were as follows: (1) there were more than three factors unanswered; (2) all the factors of importance were answered the same; and (3) the answers displayed an obvious pattern. A total of 136 of the questionnaires were effective, with a recovery rate and an effective rate of 95.86% and 97.84%, respectively. The analysis results showed that the correlation coefficient for the two tests was 0.83, indicating that the retest reliability coefficient was ideal (a retest reliability coefficient above 0.70 is considered to be optimal). The overall Cronbach α was 0.92, larger than 0.80, indicating that the questionnaire had high reliability. Factor analysis showed that the KMO value was 0.858, indicating that the selected competency elements were suitable for factor analysis, and the questionnaire had high structural validity.

### 2.4. Statistical Analysis

This study calculated Cronbach’s α to test the reliability of the model and employed the structural equation model to test the structural validity of it. In the structural equation model, since the competency level was an exogenous latent variable and the competency dimension was an endogenous latent variable, the maximum likelihood estimate (MLE) was used for parameter estimation. The evaluation of the model was, to be precise, the evaluation of the fitting degree of the data [31], as a result of which the goodness-of-fit index of the CMIN (chi-square value)/DF (degrees of freedom) [32] was used to evaluate the goodness of fit of the model. The data were processed with Microsoft Excel 2017 (Microsoft, Redmond, Washington, DC, USA) and analyzed with SPSS 21.0 (IBM, Armonk, NY, USA).

### 2.5. Ethical Issues

Informed consent forms had been completed before the survey. All the participants were provided with written and verbal information about the content and gave written informed consent to participate. The experts’ responses were protected throughout the Delphi consultations, and the participants had the right to refuse to answer questions or withdraw from the study whenever they wanted to. The study had no privacy or ethical implications.

## 3. Results

### 3.1. The Results of the Delphi Method

Two rounds of expert consultation suggested that the expert opinions were relatively concentrated. The coefficient of variation of the first round of the consultation experts’ evaluation of the competency elements ranged from 0.09 to 0.23, and that of the second round ranged from 0.08 to 0.22. The degree of the fluctuation of the second round decreased, and the opinions were relatively concentrated. According to the chi-square test, the *p* values of the two rounds of consultation were less than 0.05, which indicated that the expert opinions were coordinated and the evaluation results were satisfactory, so the expert consultation could be terminated.

After the first round of expert consultation, four elements (“optimism”, “command and monitoring”, “authorization”, and “honesty and integrity”) were deleted. Moreover, one element (“learning ability”) was added, since it could reflect nurse managers’ ability to renew knowledge continuously in the context of the rapid update of nursing knowledge and skills. Additionally, the definitions of four elements (“quality and safety awareness”, interpersonal communication”, “deductive ability”, and “inductive ability”) were modified. After the second round, the element with a mean less than 3.94 and variance larger than 0.16 (“career interest”) was deleted. Compared with the first round, the mean of each element increased, the standard deviation decreased, the boundary value increased, and the coefficient of variation decreased in the second round. The changes in these indicators demonstrated that the dispersion degree of the expert opinions in the second round decreased, the coordination degree improved, and the opinions tended to be centralized. Therefore, the expert consultation ended and the third round of consultation was no longer carried out. Finally, 27 elements remained.

### 3.2. The Results of the Questionnaire Survey

#### 3.2.1. Characteristics of the Respondents

In the official survey, a total of 600 questionnaires were issued, and 518 of them were collected, with an effective rate of 92.67%. Due to the particularity of the professional status of nurse managers, 100% of the participants were female; 85.33% were aged between 30 and 49; 64.29% had obtained a bachelor’s degree; 92.85% had obtained a professional title of middle, sub-senior, or senior; 88.63% had work experience of 6 to 30 years; 91.12% had position ranks of nurse manager; 56.82% did not have any awards; and 67.10% had incomes of CNY 4001 to 8000. The regional distribution of the nurse managers was relatively balanced, with nurse managers from Beijing, Changchun, and Haikou accounting for 30% (Table 1).

#### 3.2.2. Descriptive Statistics of the Nurse Manager Competency Factor Questionnaire

The questionnaire included 27 competency elements with an average value of 4.49, maximum value of 4.87, and minimum value of 4.26. The maximum full-score rate was 99.35%, the minimum was 86.36%, and the average was 94.87% (Table 2). According to the principle of the critical value method, elements with average values less than 4.32 and coefficients of variation larger than 0.16 should be deleted. Therefore, “information seeking”, “influence”, “organization awareness”, and “relationship building” were deleted, and the other 23 elements were retained.

### 3.3. Factor Analysis and Dimensions

Bartlett’s test of sphericity was significant at *p* < 0.05, and the KMO value was 0.928, supporting the appropriateness of the factor-analysis technique. Four principal components were extracted from the principal component analysis and were defined as the four dimensions of the nurse manager competency model. The four components cumulatively explained 62.21% of the total variance with an acceptable result. A varimax rotation was used to transform the components into factors so as to make the data more clearly interpretable. Factors with factor loadings greater than 0.5 were extracted. All 23 factors had factor loadings above 0.5 and were retained in this study. Considering the contents of the factors under each dimension, we named the four dimensions leadership and management ability (eight elements), personal traits (six elements), professional quality (three elements), and professional ability (six elements), which reflected the competency characteristics of nurse managers in tertiary general hospitals at different levels (Table 3).

### 3.4. Reliability and Validity Analysis of the Model

#### 3.4.1. Reliability Test of the Model

The overall Cronbach α coefficient of the competency element questionnaire was 0.945, greater than 0.90, indicating that the overall reliability of the questionnaire was good. Additionally, the Cronbach α coefficient of each dimension was above 0.70, suggesting that the internal consistency of each dimension of the questionnaire was high. However, the coefficient of the correlation between the achievement motivation element and the overall questionnaire was lower than 0.50, so this element was deleted. After deleting the “achievement motivation” element, the overall Cronbach α value of the modified system was 0.946, greater than 0.90, indicating that the overall reliability of the system was good. The Cronbach α values of each dimension were 0.885, 0.865, 0.863, and 0.745, respectively, all greater than 0.70, indicating a high degree of internal consistency for each dimension of the questionnaire (Table 4). As a result, the competency element system of nurse managers in tertiary general hospitals, which contained 22 elements in four dimensions, passed the reliability test and its reliability was good.

#### 3.4.2. Validity Test of the Model

In the obtained results, the main indexes for evaluating the model fit degree, NFI, RFI, IFI, TLI, CFI, and GFI, were 0.912, 0.898, 0.933, 0.918, 0.920, and 0.906, respectively, and the values of each index were all greater than or very close to 0.90, suggesting that the model fit degree was acceptable (Table 5). The ECVI (expected cross-validity index) of the study model was 3.314, lower than that of 13.62 of the independent model, suggesting that the theoretical model could be accepted. The RMSEA (root-mean-square error of approximation) was 0.072, less than 0.08, implying a good fit. The CMIN/DF was 2.847, less than 5, indicating that the model was acceptable. Therefore, through the analysis of the statistical indicators of the goodness of fit of the above models, the overall fitting effect of the nurse manager competency model of tertiary general hospitals was relatively ideal. Based on the coefficient of the correlation between the above element dimensions and the elements, it could be concluded that the structural system of competency elements constructed by the research model had good structural validity.

### 3.5. Comparison of Traditional Selection and Competency-Based Selection

The nurse manager competency model of tertiary general hospitals can be used to set standards for talent selection. In the traditional method, candidates are scored subjectively from four first-level dimensions, so candidates can only receive first-level index scores and total scores. This kind of fuzziness cannot reflect the fairness and science of selection, and the candidates cannot improve their competence. The selection method based on the competency model can make up for this deficiency and provide guidance for the development of candidates (Table 6). The elements of the competency model can clearly explain the calculation process for the scores to the candidates, which increases the acceptance of the scores by the candidates. At the same time, candidates can strengthen and cultivate the competency elements according to the standard requirements of the head nurse position, so as to adapt to the needs of the post and their own career development.

## 4. Discussion

### 4.1. The Features of the Nurse Manager Competency Model of Tertiary General Hospitals

Firstly, our findings suggest that leadership and management ability occupies a critical part of the nurse manager competency model, which is consistent with prior studies [33]. Besides thinking skills and personal disciplines, this model adds interpersonal communication and learning ability as key elements, requiring nurse managers to master a greater variety of leadership skills. As nurse managers, it is of necessity to have effective leadership to achieve positive patient outcomes and department goals [34]. In addition, nurse managers must have strong learning abilities to play an exemplary role in clinical work and management.

Secondly, this model lays emphasis on the occupational qualities of nurse managers. When nurse managers provide healthcare services, ensuring the quality and safety of nursing care must be considered [35]. Among all the elements of occupational qualities, quality and safety awareness is the foundation, while responsibility and initiative further guarantee the quality and safety of nursing. Prior studies suggest that clinical expertise is considered an important domain of nurse competency and usually covers a wide range of clinical knowledge, clinical skills, infection-control practice, etc. [33,36].

This study also included professional capacities but simplified the elements: clinical nursing professional skills, professional knowledge, and teaching ability. The professional skills and knowledge of clinical nursing are the basis, while teaching ability affects the training levels of nurse managers for the nursing team of the department. This change reflects that this model focuses more on management skills and personalities besides mere clinical practice.

Finally, personal traits reflect the deep characteristics unique to individuals, which are indispensable in the nurse manager competency model since nurse managers lead by example and train their staff to achieve qualified nursing service standards [37].

### 4.2. The Comparative Analysis of Nurse Manager Competency Models

There are similarities and differences between nurse manager competency models in relevant articles published between 2010 and 2020 and the nurse manager competency model of tertiary general hospitals in China [37]. Compared to these models, the dimensions of the nurse manager competency model of tertiary general hospitals in China are similar, but the details differ (four dimensions and 22 factors). The details of the leadership and management dimension and professional capacities dimension are exactly the same.

In terms of differences, the factors of some dimensions are different. Firstly, the numbers of factors under the leadership and management dimension are different. In this study, we identified seven factors to measure different forms of the leadership and management dimension according to the nurse regulations in China, which include leadership, interpersonal communication, self-control, learning ability, deductive ability, inductive ability, and group decision making. In the case of emergencies, nurse managers need to use their own inductive and deductive abilities and work with teams to discover the existing problems, causes, and solutions, during which thinking, communication, and self-control abilities are important.

In addition to leadership and management, nurse managers need to have an awareness of quality and safety, ensuring that patient safety is fundamental to healthcare delivery [38]. Hospitals are multi-system organizations; the team cooperation ability and “patient-centered” service consciousness should be considered.

Finally, the personal traits dimension is emphasized in our study, reflecting the deep characteristics unique to individuals, which includes six factors. Nurse managers with high organizational commitment will have more sense of value and belonging. Attention to details will play a great role in reducing nursing risk and improving patient satisfaction. Self-confidence helps nurse managers to withstand pressure, take responsibility, and cope with challenges. Physical quality is the basis of intensive nursing and management work. Good adaptability is an essential quality for nurse managers to deal with all kinds of emergency situations. Nurse managers with strong self-evaluation abilities can clearly recognize their strengths and weaknesses, providing a basis for the improvement of their work [37].

### 4.3. The Application of The Nurse Manager Competency Model

The nurse manager competency model can be applied in multiple scenarios. First, it can be used to set standards for talent selection. Through behavioral event interviews, the performance of nurse managers can be evaluated by scenario-simulation tests. Based on the competency index and behavior description in this model, hospitals can develop a unified talent evaluation standard to investigate the degree of conformity between a nurse manager’s abilities and this model. Second, the model provides a reference for hospitals with which to make training plans and career plans for nurse managers. After detecting the competency gap, hospitals can design specific vocational training programs for nurse managers and make position-transfer arrangements. Third, since the model is reflective of a nurse manager’s comprehensive abilities and the current state of healthcare, it can be employed as a tool of performance assessment [39]. A performance-evaluation system based on both traditional assessment standards and the competency model are inclined to be more equitable, systematic, and scientific. Finally, as the core of nursing management changes from experience management to scientific management [14], an advanced nurse manager competency model can contribute to the update of nursing philosophy.

## 5. Conclusions

This study examined the nurse manager competency model of tertiary general hospitals in China. This model is an instrument consisting of four dimensions and 22 competency elements with fair reliability and validity, and fully reflects the characteristics of nurses in Chinese public hospitals. The methods adopted in this study can provide reference for other countries to establish job evaluation factors for the selection of new nurse managers.

## Figures and Tables

**Table 1:** Characteristics of the study participants.

	Characteristics	Frequency	Percent
Gender	Female	518	100.00
	Male	0	0.00
Age	30 and below	2	0.39
	30–39	246	47.49
	40–49	196	37.84
	50 and above	74	14.29
Educational level	Associate degree and below	49	9.46
	Bachelor’s degree	333	64.29
	Master’s degree	135	26.06
	Doctoral degree	1	0.19
Professional title	Junior	37	7.14
	Middle	316	61.00
	Sub-senior	148	28.57
	Senior	17	3.28
Working years	1–5	13	2.60
	6–10	67	12.99
	11–15	89	17.21
	16–20	123	23.70
	21–25	124	24.03
	26–30	56	10.71
	30 and above	45	8.77
Position rank	Nursing staff	26	5.02
	Department nurse manager	20	3.86
	Nurse manager	472	91.12
Whether had awards	Yes	224	43.18
	No	294	56.82
Income	2000–4000	24	4.56
	4001–6000	177	34.20
	6001–8000	170	32.90
	8001–10,000	110	21.17
	10,000 and above	37	7.17

**Table 2:** Scores of importance of competency elements.

Number	Competency Element	Mean	Standard Error	Full-Score Rate (%)	Coefficient of Variation
1	Achievement motivation	4.39	0.64	92.86%	0.15
2	Quality and safety awareness	4.87	0.44	99.35%	0.09
3	Initiative	4.67	0.57	98.05%	0.12
4	Information seeking	4.30	0.67	89.61%	0.16
5	Interpersonal communication	4.62	0.67	96.10%	0.14
6	Patient service awareness	4.62	0.58	98.05%	0.13.
7	Influence	4.29	0.79	87.66%	0.18
8	Organization awareness	4.26	0.80	86.36%	0.19
9	Relationship building	4.28	0.73	87.66%	0.17
10	Developing others	4.61	0.56	99.35%	0.12
11	Teamwork and cooperation	4.78	0.50	99.35%	0.10
12	Leadership	4.65	0.57	98.70%	0.12
13	Group decision making	4.54	0.61	97.40%	0.13
14	Deductive ability	4.33	0.67	90.91%	0.15
15	Inductive ability	4.34	0.65	92.21%	0.15
16	Professional knowledge	4.52	0.67	93.51%	0.15
17	Clinical nursing skills	4.60	0.61	98.05%	0.13
18	Teaching ability	4.44	0.61	96.75%	0.14
19	Learning ability	4.55	0.54	99.35%	0.12
20	Self-control	4.57	0.62	96.10%	0.14
21	Self-confidence	4.40	0.64	96.10%	0.15
22	Adaptability	4.38	0.62	95.45%	0.14
23	Organizational commitment	4.35	0.66	94.16%	0.15
24	Attention to details	4.38	0.68	92.21%	0.15
25	Physical health	4.38	0.68	93.51%	0.15
26	Self-evaluation	4.48	0.62	96.75%	0.14
27	Responsibility	4.76	0.56	98.05%	0.12

**Table 3:** Loading table for competency elements after rotation.

	Component			
	1	2	3	4
Achievement motivation	0.586			
Interpersonal communication	0.653			
Leadership	0.566			
Group decision making	0.562			
Deductive ability	0.719			
Inductive ability	0.662			
Learning ability	0.504			
Self-control	0.504			
Self-confidence		0.525		
Adaptability		0.675		
Organizational commitment		0.658		
Attention to details		0.740		
Physical health		0.694		
Self-evaluation		0.683		
Quality and safety awareness			0.779	
Initiative			0.572	
Patient service awareness			0.601	
Developing others			0.576	
Teamwork and cooperation			0.690	
Responsibility			0.503	
Professional knowledge				0.530
Clinical nursing skills				0.707
Teaching ability				0.696

**Table 4:** Reliability test of competency elements.

Elements	Cronbach α	Degree of Correlation	Cronbach α after Deleting This Element
Interpersonal communication	0.946	0.639	0.943
Leadership		0.521	0.944
Group decision making		0.636	0.942
Deductive ability		0.661	0.942
Inductive ability		0.739	0.941
Learning ability		0.685	0.942
Self-control		0.686	0.942
Self-confidence		0.614	0.943
Adaptability		0.697	0.941
Organizational commitment		0.698	0.941
Attention to details		0.616	0.943
Physical health		0.569	0.943
Self-evaluation		0.628	0.942
Quality and safety awareness		0.663	0.942
Initiative		0.681	0.942
Patient service awareness		0.621	0.942
Developing others		0.661	0.942
Teamwork and cooperation		0.572	0.943
Responsibility		0.669	0.942
Professional knowledge		0.623	0.943
Clinical nursing skills		0.685	0.942
Teaching ability		0.638	0.942

**Table 5:** Goodness of fit of nurse manager competency model of tertiary general hospitals.

Model	NFI	RFI	IFI	TLI	CFI	GFI	ECVI	RMSEA	CMIN/DF
Research model	0.912	0.898	0.933	0.918	0.920	0.906	3.314	0.072	2.847
Saturated model	1.000		1.000		1.000	1.000	3.595	0.215	
Independent model	0.000	0.000	0.000	0.000	0.000	0.205	13.620	0.072	11.962

**Table 6:** Comparison of traditional selection and competency-based selection.

Items	Traditional Selection	Competency-Based Selection
Base of selection	job analysis	hospital values and strategic targets, job analysis
Person–post matching	static matching	dynamic matching
Quality of candidates	able to complete work	competent in work
Contract	labor contract	labor and psychological contract
Evaluation index	knowledge and skills	competency
Training of evaluation team	little training	scientific and systematic training
Evaluation procedure	no standard procedure	standardized evaluation
Evaluation method	qualitative	qualitative and quantitative

## Data Availability

The data presented in this study are available on request from the corresponding author. The data are not publicly available due to the information contained that could compromise the privacy of the research participants.
